# Dementia and Sandwiched Caregiving: Effects on Caregiver and Older Adult Psychological Well-Being

**DOI:** 10.1093/geroni/igaf122.2076

**Published:** 2025-12-31

**Authors:** Natasha Nemmers, Sarah Patterson, Yuchen Zhang, Virginia Gallagher

**Affiliations:** Wayne State University, Detroit, Michigan, United States; University of Michigan, Ann Arbor, Michigan, United States; University of Pittsburgh School of Nursing, Pittsburgh, Pennsylvania, United States; University of Virginia, Charlottesville, Virginia, United States

## Abstract

The population of sandwiched caregivers—those supporting both an older adult and a minor child—is increasing yet understudied particularly regarding dementia care. Existing research on this group often focuses on adult children, neglecting other caregivers (e.g., neighbors or grandchildren). This study examines psychological well-being in sandwiched versus non-sandwiched caregivers and their older adult care recipients by dementia status [person living with dementia (PLWD) versus non-PLWD]. Data from 1,514 caregivers and 1,109 older adults were analyzed using the 2022 National Health and Aging Trends Study and National Study of Caregiving. Among caregivers, 253 had a child under 18: 99 cared for PLWD and 154 for non-PLWD. Non-sandwiched caregivers (n = 1,261) included 524 caring for PLWD and 737 non-PLWD. Multivariate linear regression models assessed both caregiver and older adult well-being, adjusting for demographic and care-related covariates. Models were weighted and adjusted for NHATS/NSOC’s complex survey design. For caregiver well-being, sandwiched caregivers had significantly higher well-being (B = 1.85, p<.01) relative to non-sandwiched. However, care-recipient dementia status was not significantly associated with caregiver well-being. The interaction between dementia status and being sandwiched was significant (B=-2.18, p<.01), indicating a reduced well-being advantage for sandwiched caregivers of PLWD. Among older adults, PLWD had significantly lower well-being than non-PLWD (B=-7.36, p<.001). These findings suggest that caregivers with children may experience protective benefits if their older adult care-recipient does not have dementia, whereas dementia negatively impacts well-being among both members of the caregiving dyad. Future research should focus on tailored strategies to improve dyadic well-being in these complex caregiving situations.

